# Commentary on “Epigenetically Altered T Cells Contribute to Lupus Flares”

**DOI:** 10.33696/immunology.1.004

**Published:** 2019

**Authors:** Bruce Richardson

**Affiliations:** Department of Internal Medicine, University of Michigan Medical School, Ann Arbor, MI 48103-2200, USA

The recently published manuscript entitled “Epigenetically Altered T Cells Contribute to Lupus Flares” summarizes recent advances in our understanding of how the environment alters the immune system to cause flares of systemic lupus erythematosus (SLE) in genetically predisposed people, and why it affects women approximately 9 times more often than men [1]. SLE is characterized by the formation of autoantibodies to “self” antigens such as DNA. Lupus typically develops at somewhere between 15 and 40 years of age [1], then follows a chronic relapsing course with flares triggered by environmental agents that cause inflammation, such as infections and sun exposure [2]. How the environmental agents alter the immune system to cause lupus flares though has long been obscure. Certain drugs though, and procainamide and hydralazine in particular, also cause lupus-like autoimmunity in genetically predisposed people, and have provided an approach to determine how exogenous agents can alter immune responses to cause lupus flares.

The manuscript summarizes how in vitro studies on the epigenetic regulation of gene expression in T lymphocytes can alter their function to cause lupus flares. Briefly, each nucleated cell in the human body contains approximately 3 meters of DNA, tightly packaged in the nucleus as chromatin, consisting of the DNA wrapped around a core of 8 histone proteins. The tightly packaged DNA is inaccessible to the transcription factors, and the chromatin structure must be locally “opened up” to allow the relevant transcription factors to bind and allow mRNA synthesis to proceed. The cell promotes the condensation of DNA into a transcriptionally repressive configuration by methylating dC bases located in CpG pairs. This allows the binding of methylcytosine binding proteins to the methylated regions, which then attract the chromatin inactivation complex which promotes the condensation of the DNA into a tightly packaged chromatin structure. However, the DNA methylation patterns must be replicated accurately every time a cell divides, and it is at this point where DNA methylation becomes sensitive to environmental influences. Inhibiting methylation of the newly synthesized DNA strand allows expression of those genes previously suppressed by DNA methylation, and for which the cell expresses the necessary transcription factors.

Early studies demonstrated that treating CD4^+^ T cells with the covalent DNA methyltransferase inhibitor 5-azacytidine (5-azaC) converts antigen specific CD4^+^ T cells into autoreactive cells which respond to self class II MHC molecules on antigen presenting cells without added antigen, thus becoming autoreactive [3]. The pathologic significance of the autoreactivity was tested by treating polyclonal or cloned, antigen specific CD4^+^ murine T cells with 5-azacytidine (5-azaC) then injecting the treated cells into syngeneic recipients. Mice receiving the treated, but not untreated T cells developed lupus-like autoimmunity [4]. Further, similar epigenetically altered CD4^+^ T cells were found in lupus patients, and the number of the epigenetically altered cells was directly proportional to flare severity. In addition, the two drugs which cause drug-induced lupus most frequently, procainamide and hydralazine, were found to be DNA methylation inhibitors [5]. The studies reviewed in the article entitled “Epigenetically Altered T Cells Contribute to Lupus Flares” in this issue of Cells summarize these studies, and how they led to studies demonstrating similar changes in a subset of CD4^+^ T cells from patients with active lupus. Together the experiments indicate a mechanism by which environmental agents that cause inflammation can trigger lupus flares.
